# Oxidative Stress, Chronic Inflammation, and Amyloidoses

**DOI:** 10.1155/2019/6024975

**Published:** 2019-09-08

**Authors:** Arkadiusz Orzechowski, Anna Cywińska, Agueda A. Rostagno, Federica M. Rizzi

**Affiliations:** ^1^Department of Physiological Sciences, Warsaw University of Life Sciences (SGGW), Nowoursynowska 159, 02-776 Warsaw, Poland; ^2^Department of Pathology and Veterinary Diagnostics, Warsaw University of Life Sciences (SGGW), Nowoursynowska 159, 02-776 Warsaw, Poland; ^3^Department of Pathology, New York University School of Medicine, 550 First Avenue, MSB 556, New York, NY 10016, USA; ^4^Dipartmento di Medicina a Chirurgia, Universita degli Studi di Parma, Via Volturno 39/E, Parma, Italy

Amyloid diseases are part of an emerging complex group of chronic and progressive entities collectively known as disorders of protein folding. For reasons still under active investigation, soluble proteins normally found in interstitial or biological fluids change their conformation and form poorly soluble molecular assemblies that accumulate as extracellular fibrillar aggregates within the parenchyma and blood vessels of different organs. Once formed, these amyloid fibrils that typically exhibit a *β*-sheet-rich secondary structure are highly resistant to proteolytic degradation, a characteristic that impairs their effective physiologic removal and leads to their tissue accumulation inducing local hypoxia and cellular damage with overall organ dysfunction and, eventually, death.

The molecular pathogenic mechanisms associated with amyloid diseases are complex and involve cross-talk among different cell populations and cellular pathways. Mounting evidence points out to inflammation-related mechanisms and oxidative stress as associated burden for amyloidosis. In spite of multiple studies, it still remains to be elucidated whether oxidative stress is a key contributor to the disease pathogenesis and progression or whether—on the contrary—it is a mere consequence of the cellular responses elicited by the presence of amyloid deposits and the concomitant inflammatory processes affecting injured tissue. This special issue provides an updated view on the role of oxidative stress and inflammation-related cellular pathways to the etiopathogenesis, progression, and treatment strategies of amyloid diseases.

One of the papers of this special issue, “Hypoxia and Inflammation as a Consequence of *β*-fibril Accumulation: A Perspective View for New Potential Therapeutic Targets,” addresses the link between local hypoxia and the induction of chronic long-lasting inflammation in the context of the tissue accumulation of the *β*-sheet-rich amyloid deposits. The authors propose that the induction of cell death mechanisms in conjunction with local tissue hypoxia associated with the amyloid accumulation constitutes important events for the pathogenesis and progression of amyloidosis. The authors hypothesize that molecules released by necrotic cells activate inflammatory responses through Damage-Associated Molecular Patterns (DAMPs) and evoke activation of HIF-1*α*/NF-*κ*B-dependent pathways suggesting that modulation of these cellular paths may constitute new therapeutic strategies. Another manuscript of the special issue focuses on the role of oxidative stress for the process of amyloid formation. The authors of “Suppression of Mouse AApoAII Amyloidosis Progression by Daily Supplementation with Oxidative Stress Inhibitors” examined the effect of the nonspecific reactive oxygen species (ROS) scavenger tempol and the NADPH oxidase inhibitor apocynin on the process of amyloidogenesis using a mouse model of AApoAII amyloidosis. Their results provide evidence that oxidative stress is involved in the progression of systemic amyloidosis but indicate that, although oxidative stress inhibitors ameliorated amyloid deposition, their use was unable to completely block the progression of amyloidosis. The paper entitled “High levels of *β*-Amyloid, Tau, and Phospho-Tau in Red Blood Cells as Biomarkers of Neuropathology in Senescence-Accelerated Mouse” describes a study conducted on the Senescence-Accelerated Mouse-Prone, SAMP8, a validated model of Alzheimer's disease (AD) with the aim of establishing novel sensitive disease biomarkers. The study illustrates the increasing brain accumulation of amyloid-beta (A*β*) as well as total and phosphorylated tau concomitant with the elevated levels of the inflammatory marker interleukin-1*β*. The brain accumulation kinetics parallels findings in red blood cells suggesting these proteins as putative peripheral AD biomarkers. The authors of the original manuscript “Syk and Hrs Regulate TLR3-Mediated Antiviral Response in Murine Astrocytes” examined the regulation of the innate immune response-related Toll-like receptor 3 (TLR3) signaling in the astrocytic cell line C8-D1A following stimulation with a viral dsRNA mimetic (Pathogen-Associated Molecular Patterns (PAMPs)). The study uncovers a relationship between TLR3 and the endosomal sorting complex required for transportation-0 (ESCRT-0), pointing out to the spleen tyrosine kinase (Syk) as a dsRNA-activated kinase mediating TLR3 signaling and the concomitant induction of IFNs and proinflammatory gene expression. The paper “Nitric Oxide Influences HSV-1-Induced Neuroinflammation,” aimed to elucidate the role of NO in neuroinflammation and neurodegeneration based on the use of *in vitro* models of herpes simplex virus (HSV-1) infection of neuronal and glial cell cultures as well as intranasal viral infection of BALB/c mice. The authors describe a differential response to NO and HSV-1 infection for neuronal, microglial, and astrocytic cells as well as in the concomitant production of IFN-alpha (IFN-*α*) and proinflammatory cytokines CXCL9 and CXCL10. Presented results help elucidate the molecular roles of NO and NO-related signaling during HSV-1-induced neuroinflammation and neurodegeneration and indicate for the first time the existence of a link between Fas signaling due to neuroinflammation and nitrosative stress during HSV-1 infection.

Overall, the special issue highlights the contribution of oxidative stress and chronic inflammation to the pathobiology of amyloid-associated diseases. The papers compiled in this special issue widen the current knowledge of the molecular mechanisms involved in the etiology, progression, and accumulation of *β*-sheet-enriched deposits in infectious and noninfectious forms of amyloidosis.

